# Structural and Developmental Disparity in the Tentacles of the Moon Jellyfish *Aurelia sp*.*1*

**DOI:** 10.1371/journal.pone.0134741

**Published:** 2015-08-04

**Authors:** David A. Gold, Nagayasu Nakanishi, Nicholai M. Hensley, Kira Cozzolino, Mariam Tabatabaee, Michelle Martin, Volker Hartenstein, David K. Jacobs

**Affiliations:** 1 Department of Ecology and Evolutionary Biolology. University of California Los Angeles, Los Angeles, California, United States of America; 2 Department of Molecular, Cell, and Developmental Biology. University of California Los Angeles, Los Angeles, California, United States of America; UC Irvine, UNITED STATES

## Abstract

Tentacles armed with stinging cells (cnidocytes) are a defining trait of the cnidarians, a phylum that includes sea anemones, corals, jellyfish, and hydras. While cnidarian tentacles are generally characterized as structures evolved for feeding and defense, significant variation exists between the tentacles of different species, and within the same species across different life stages and/or body regions. Such diversity suggests cryptic distinctions exist in tentacle function. In this paper, we use confocal and transmission electron microscopy to contrast the structure and development of tentacles in the moon jellyfish, *Aurelia species 1*. We show that polyp oral tentacles and medusa marginal tentacles display markedly different cellular and muscular architecture, as well as distinct patterns of cellular proliferation during growth. Many structural differences between these tentacle types may reflect biomechanical solutions to different feeding strategies, although further work would be required for a precise mechanistic understanding. However, differences in cell proliferation dynamics suggests that the two tentacle forms lack a conserved mechanism of development, challenging the textbook-notion that cnidarian tentacles can be homologized into a conserved *bauplan*.

## Introduction

**Fig 1:**
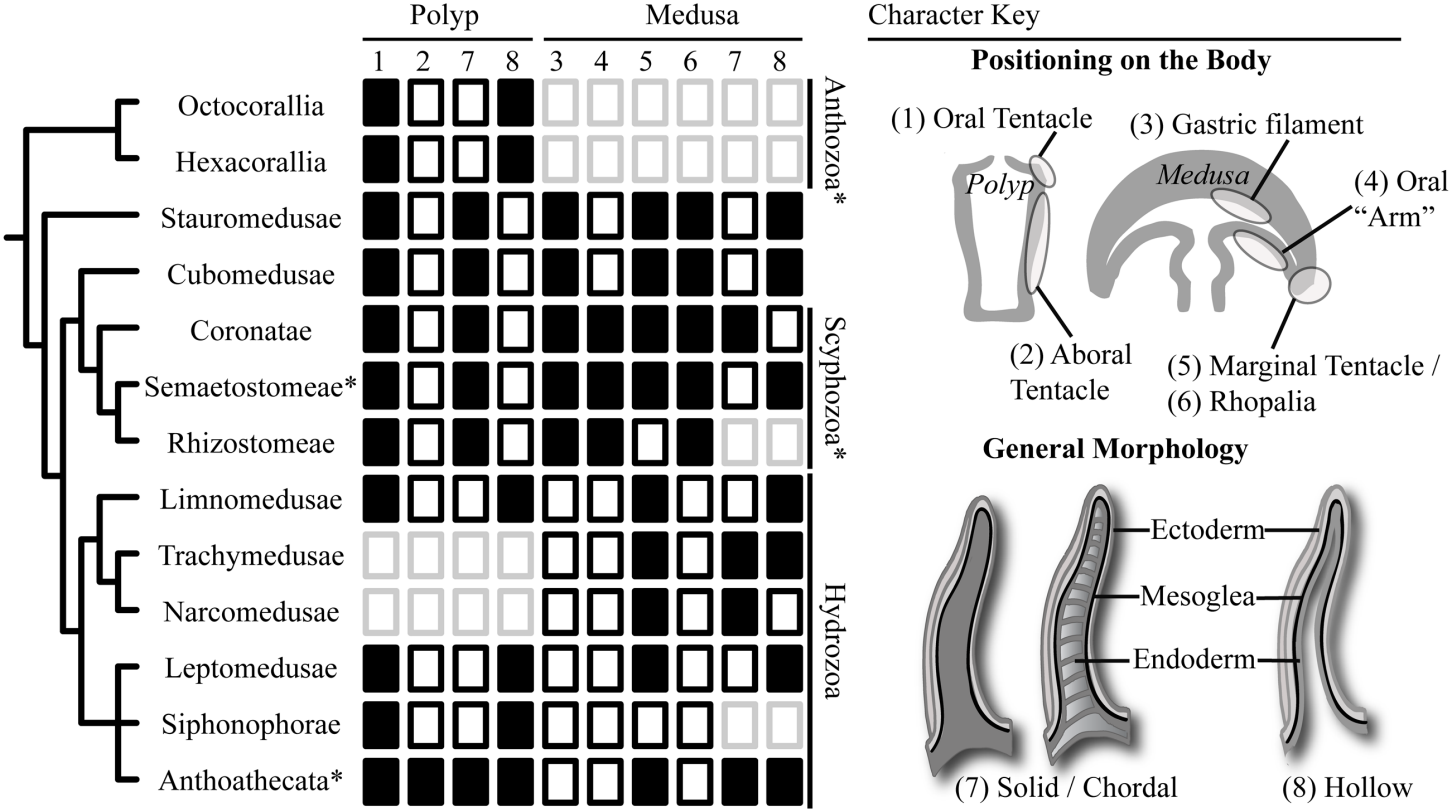
Diversity and distribution of tentacle-like structures across the Cnidaria. (Left) A consensus phylogeny of the Cnidaria (based on [9,10,14], see [15] for an opposing topology). Clades marked with an asterisk have been deemed paraphyletic in one or more molecular phylogenetics study. (Middle) The distribution of characters across the cnidarian tree: black box = character present; white box = character absent; grey box = character not applicable (i.e. the relevant life stage or tentacle type does not exist). Character states adapted from [10], with the exception of oral arms, where we used a more lenient definition for character presence. (Right) A visual key summarizing character states.

The Cnidaria (corals, sea anemones, jellyfish, and hydroids) encompasses more than 10,000 species [1] with a wide range of morphologies, ecologies, and life histories. This diversity is generated in large part from the tentacles (Fig 1), which vary significantly in form, positioning on the body axis, and cnidocyte composition and arrangement [2–6]. Tentacles are disparate enough to be a common component of phylogenetic analyses [5,7–12], and there exists significant disagreement about the homology of various tentacle types [6,7,13,14], although the criteria for determining homology is rarely discussed or defended.

As part of our continued research into the development of *Aurelia species 1* (class Scyphozoa, order Semaeostomeae / Discomedusae, species *sensu* [16]), we used confocal and transmission electron microscopy to compare the anatomy of the two major tentacles types: the oral tentacles of the polyp, and the marginal tentacles of the medusa. The microanatomy of *Aurelia* tentacles has been studied previously, [17–22], but this is the first time that the two have been analyzed in a comparative manner. Ultimately, we found that oral and marginal tentacles are dissimilar in both cellular construction and mode of development. Many of these differences could represent evolutionary or morphological constraints, or they could be adaptive biomechanic solutions to divergent feeding strategies. However, fundamental differences between oral and marginal tentacle growth challenge the hypothesis that these structures can be homologized as part of a conserved cnidarian *bauplan* (i.e. that the medusa is essentially an upside-down polyp). We expect that this project will set the groundwork for future research on the mechanics and developmental genetics of *Aurelia* tentacles, and hope that it will promote a more nuanced interpretation of research regarding the evolution and function of tentacles across the Cnidaria.

## Materials and Methods

Animals were raised in the aquarium at UCLA, and the Cabrillo Marine Aquarium in San Pedro, California. All animals were relaxed in 7.3% MgCl_2_ before being fixed in 4% formaldehyde for 1 hour at room temperature. Cell proliferation was assayed by incubating live animals for two hours in 100 μM 5-ethynyl-2’-deoxyuridine (EdU) at room temperature, followed by relaxation and fixation. Fixed animals were then prepared using the standard protocol for the Click-iT EdU Alexa Fluor 488 Flow Cytometry Assay Kit (Invitrogen, cat# C35002).

Primary antibodies used for this study include those against acetylated tubulin (“Atub”; mouse, 1:1,000, Sigma), tyrosinated tubulin (“Ttub”; mouse, 1:800, Sigma), FMRFamide (“FMRF”; rabbit, 1:500 dilution, US Biological), Gonadotropin-releasing hormone (“GnRH”; rabbit, 1:500, Sigma), and a squid-specific opsin (rabbit, 1:1000, from Laura J. Robles, California State University, Dominguez Hills). The GnRH gene (*GnRH1*) does not appear to be present in *Aurelia*, based on our unpublished genomic and transcriptomic data. However, this particular antibody has been previously shown to cross-react with cnidocyte capsules in *Aurelia* planula larvae [21]. We subsequently refer to this antibody as a “capsule” (“Cap”) marker in the rest of the text. Similarly, the opsin antibody does not specifically bind to *Aurelia* opsin proteins, but consistently demarcates cell membranes. We subsequently refer to this antibody as a “membrane” (“Mem”) marker. Following primary antibody staining, animals were washed in PBSTr (PBS plus 0.3% Triton X-100) for 2 hours and blocked in 3% normal goat serum for an hour at room temperature (20–25°C). Secondary antibodies used for this study include AlexaFluor 555, AlexaFluor 488, and AlexaFluor 647 (Invitrogen). Nuclei were labeled using TO-PRO-3 Iodide (Invitrogen) or Sytox (Invitrogen). Filamentous actin was labeled using phalloidin conjugated to AlexaFluor 568. Specimens were incubated with fluorescent dyes together with secondary antibodies overnight at 4°C. The specimens were washed in PBSTr for 2 hours at room temperature (20–25°C) and were mounted in ProLong Gold (Invitrogen). Slides were viewed on a Zeiss Imager.M2 Confocal Microscope, and digital stacks were manipulated using ImageJ.

For transmission electron microscopy (TEM), animals were relaxed in 7.3% MgCl_2_ before being fixed in 4% paraformaldehyde and 2.5% glutaraldehyde for 24 h at 4°C, and then stained with 1% osmium tetroxide for 1 hour at 4°C. Animals went through an acetone dehydration series (50%, 70%, 96%, and 100% acetone), followed by an epon series (1:3 epon–acetone, 2:2, 3:1, overnight evaporation to 100%), before being transferred into plastic molds for polymerization at 60°C for 16 h. The epon blocks were submitted to the UCLA Microscopic Techniques Laboratory for sectioning, and were examined using a JEOL 100CX transmission electron microscope.

## Results

### General morphology of the polyp oral tentacle

**Fig 2:**
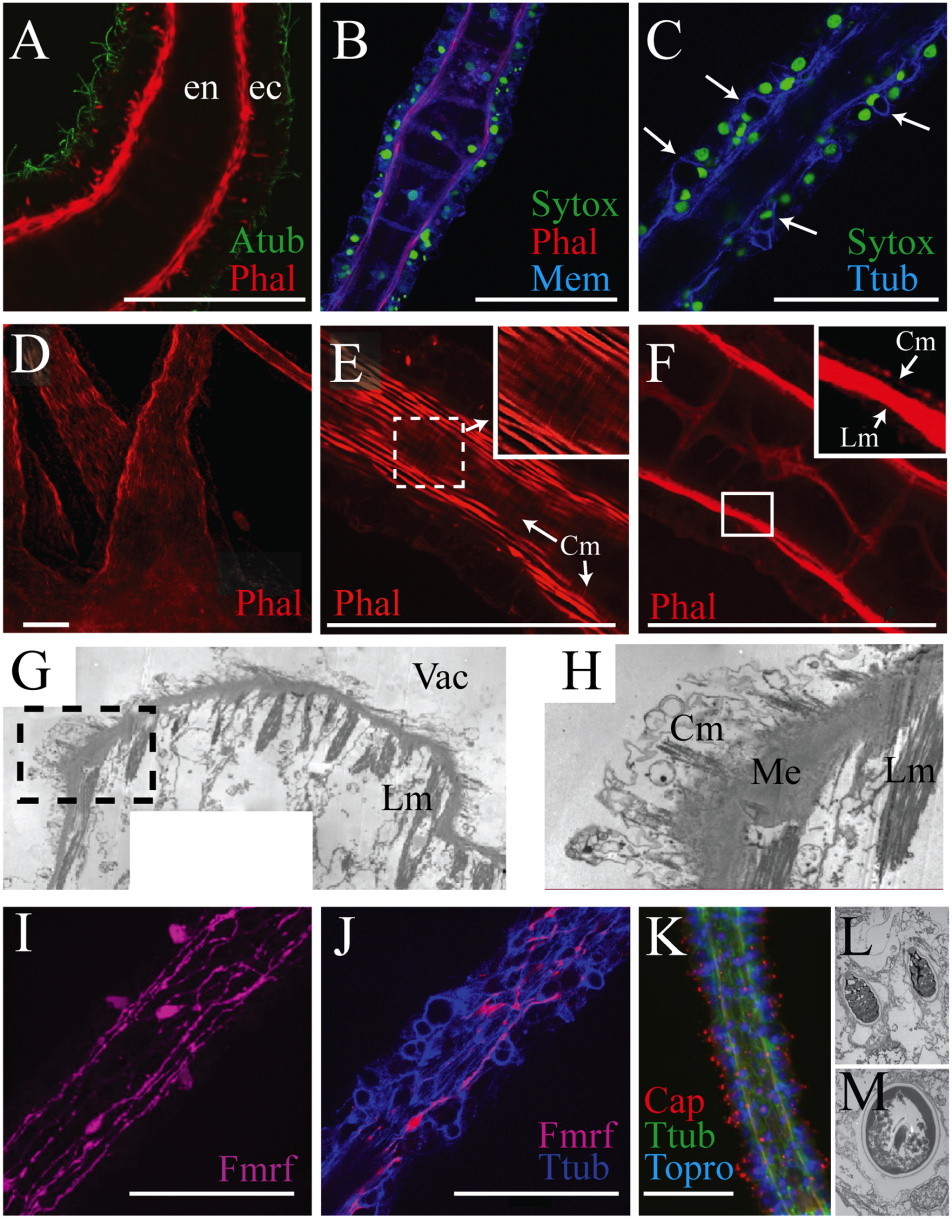
Morphology of the polyp oral tentacle in *Aurelia sp*.*1*. All scale bars represent 50 μm. (A) Longitudinal section of tentacle, revealing the morphology and distribution of ectodermal (ec) and endodermal (en) cells. (B) A similar image showing the distribution of nuclei in the tentacle. Note the row of large, vacuolated cells in the endoderm. (C) Longitudinal section demonstrating how anti-Ttub can be used to identify cnidocytes. Examples where enlarged cells (caused by the presence of the cnidocyte capsule) are co-localized with crescent-shaped nuclei are labeled with arrows. (D) Phalloidin staining at the base of a tentacle. (E) A partial stack of confocal images, revealing the circumferential myofibrils (Cm) underneath the longitudinal musculature of the epitheliomuscular cells. (F) A partial stack of confocal images deeper in the longitudinal section, which suggests that space (presumably mesoglea) separates the longitudinal and radial musculature. (G) TEM of an oblique longitudinal cut on the polyp tentacle, producing a peninsula of tissue rich in longitudinal myofibrils (Lm) situated above the vacuolated space (Vac) of an endodermal cell. (H) Close-up of the box in Fig 1G, clarifying longitudinal myofibrils (Lm), circumferential myofibrils (Cm), and mesoglea (Me). (I) Distribution of anti-FMRFamide-positive neurons and their processes. (J) Co-labeling of anti-FMRFamide and anti-Ttub. Note how anti-Ttub labels additional neural tracts that are not FMRF-positive. (K) Distribution of anti-Ttub and “capsule” positive cells. In these images, the antibody labels the apical tip of most cells; which is distinct from the cnidocyte specific expression found in the planula or medusa (see Fig 3J). (L) Longitudinal TEM section of several atrichous isorhizas (X 17,000). (M) Longitudinal TEM section of a microbasic heterotrichous eurytele (X 17,000).

In the developing polyp, four tentacles bud around the mouth; additional tentacles intercalate between developing ones, until sixteen are formed [21]. Oral tentacles are of the solid (or chordal) variety, with a single row of large, highly vacuolated cells filling the endoderm [2,18,23] (Fig 2A and 2B). The ectoderm consists primarily of epitheliomuscular cells, neurons, gland cells and cnidocytes. Based on TEM data, Chia, Amerongen, and Peteya [23] suggest that the tentacle ectoderm can be divided into a superficial epithelial and subepithelial layer. However, such layering is probably a consequence of tentacle retraction; in extended (relaxed) tentacles, nuclear staining suggests that the ectoderm is rarely more than one cell deep (Fig 2B). Epitheliomuscular cells are apically ciliated (Fig 2A), and project basal myofibrils that generate the longitudinal musculature of the tentacle (Fig 2A–2C). We did not find an asymmetric concentration of longitudinal musculature at the proximal end of the tentacle (Fig 2D), as had been reported previously [23].

Phalloidin-stained preparations often reveal a series of regularly-spaced circumferential fibers underneath the longitudinal fibers of the epitheliomuscular cells (Fig 2E and 2F), contradicting a previous report that *Aurelia* oral tentacles lack circumferential musculature [18]. A small gap between the longitudinal and circumferential fibers (Fig 2F) suggests that the two are separated by mesoglea, and that the later fibers belong to endodermal cells. TEM also supports our interpretation of circumferential fibers; as the longitudinal musculature is cut away, we find fibers of a similar quality projecting perpendicularly underneath the mesoglea (Fig 2G and 2H).

**Fig 3:**
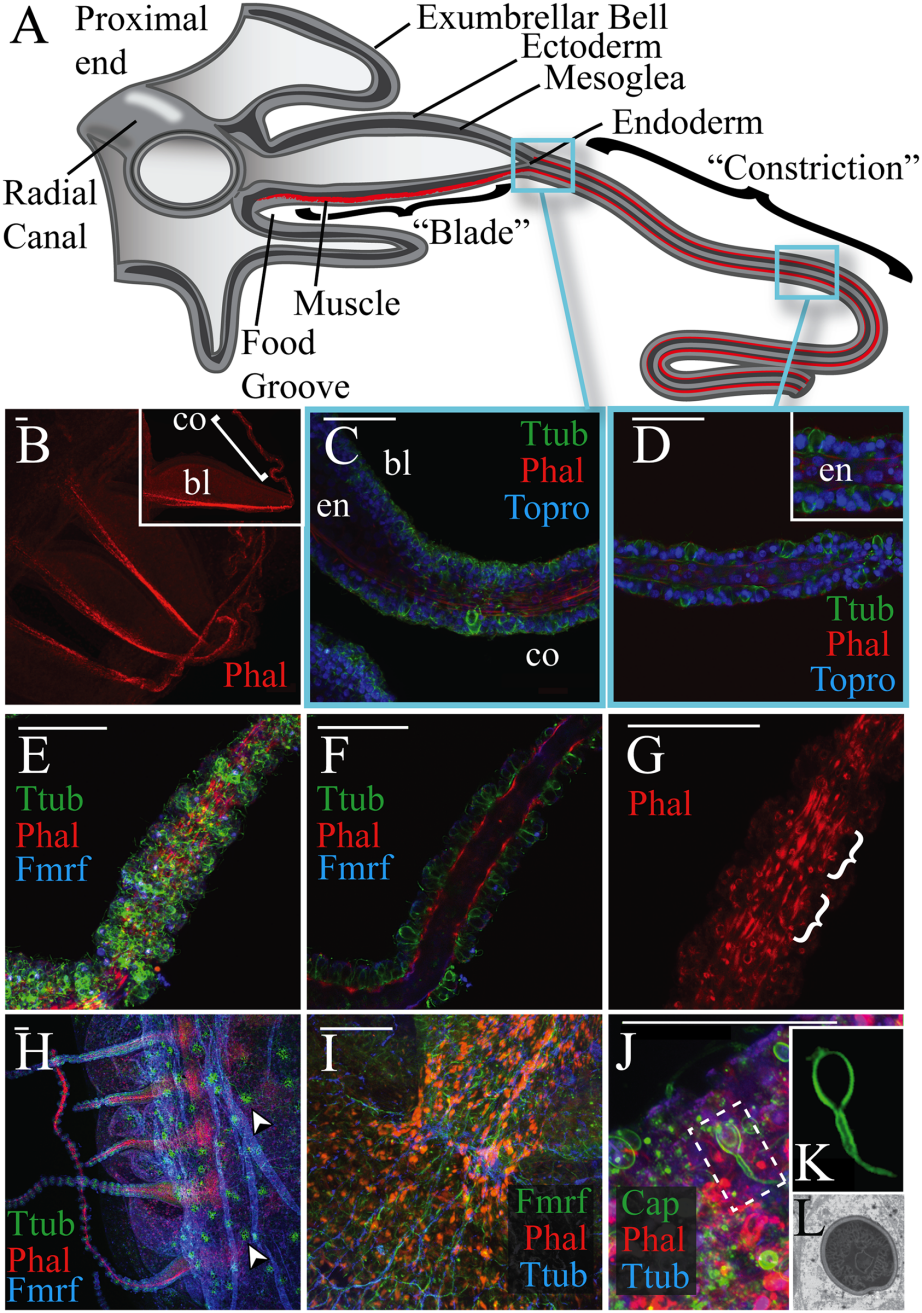
Morphology of the medusa marginal tentacle in *Aurelia sp*.*1*. All scale bars equal 50 μm. (A) Illustration of a longitudinal section of the medusa marginal tentacle in *Aurelia*. (B) Phalloidin staining of three marginal tentacles (upper left proximal, lower right distal), which elucidate the muscle chord running down the oral side of each tentacle. A single tentacle has been digitally isolated using Adobe Photoshop in the upper-right box (left proximal, bottom oral) to clarify the distinction between the proximal “blade” (bl) and distal “constriction” (co). (C) Confocal longitudinal section of the marginal tentacle, showing where the proximal “blade” changes into the radially symmetrical distal “constriction”. (D) Another confocal longitudinal section, highlighting the dense packing of endodermal (en) cells in the distal portion of the tentacle. Note the distinction between this morphology and the chordal morphology seen in the polyp (Fig 2A and 2B). (E) Z-projection illustrating the modular clusters of cells in the distal portion of the marginal tentacle. (F) A longitudinal section of Fig 3E. (G) A Z-projection of phalloidin staining in Fig 3E. Note in (F) and (G) that phalloidin bands do not appear to generate cohesive longitudinal musculature, but instead form localized musculature within each module (H) Low-magnification image of neural distribution across the medusa bell. Arrowheads indicate putative clusters of Ttub-positive neurites. (I) High magnification of the proximal-most end of the tentacle, showing FMRFamide and Ttub-positive neurites associated with the muscle chord. (J) A Z-projection revealing the high concentration of “capsule”-positive cnidocytes in the tentacle ectoderm. (K) A partially fired cnidocyte from 2J (white box), digitally isolated using Adobe Photoshop. (L) Longitudinal TEM section of a microbasic heterotrichous eurytele (X 9,000).

In the polyp tentacle, an anti-FMRFamide antibody labels ectodermal epithelial sensory cells with basal neurites, which form neural tracts along the longitudinal axis (Fig 2I and 2J). Anti-Ttub also labels neurites, demonstrating that only a subset of neurons are FMRFamide-positive, similar to what is seen in planulae [24] and ephyrae [25]. We did not find evidence for a decrease in FMRFamide-positive neural processes towards the distal tentacle tip, as has been reported in Japanese specimens of *Aurelia* [26]. Cnidocytes are scattered across the ectoderm, and can be identified using anti-Ttub combined with a nuclear stain (Fig 2C). However, our “capsule” antibody, which labels mature cnidocyte capsules in *Aurelia* planula [21], instead shows immunoreactivity at the apical tip of most cells (Fig 2H). TEM of cnidocytes demonstrates the presence of atrichous isorhizas and microbasic heterotrichous euryteles (Fig 2L and 2M), both of which can be easily identified by morphology [27,28]. Atrichous isorhizas (Fig 2L) feature a thread that completely fills the cnidocyte capsule, and coils for approximately six turns along the capsule’s length [27]. Microbasic heterotrichous euryteles (Fig 2M) are significantly larger than isorhizas and much rarer; in our sections of the polyp tentacle, we only found one eurytele for every fifteen isorhizas. Our TEM and “capsule” antibody results are in marked contrast to what we found in the medusa (see Fig 3J), and suggest that the cnidomes of polyp and medusa tentacles are largely distinct.

### General morphology of the medusa marginal tentacle

The tentacles of the *Aurelia* polyp and medusae have distinctly different ontogenies. During the metamorphosis from *Aurelia* polyp into medusa (known as strobilation), the oral tentacles of the polyp degenerate as a series of ephyra (young medusa) develop in an oral to aboral progression. Later in strobilation, new polyp tentacles regenerate below the developing ephyra. The ephyra lacks tentacles, although their sensory structures (called rhopalia) share positional and structural similarities with tentacles, and it is plausible that the two have an evolutionary relationship [13]. True medusa tentacles occur later in development through the distal and lateral expansion of eight tongue-like processes on the bell’s oral surface, which multifurcate into hundreds of small tentacles [20].

These marginal tentacles display a markedly different morphology from the oral tentacles of the polyp. As reported previously [19], marginal tentacles are broad and blade-like at the proximal end, and radically constrict towards the distal tip (the anatomy of a cross-sectioned marginal tentacle is illustrated in Fig 3A). Although most epithelial cells have basal myofibril processes, coordinated musculature in the “blade” is restricted to a thick longitudinal chord running along the oral side (Fig 3A and 3B). As the proximal portion of the tentacle constricts, all epitheliomuscular cells start forming coordinated longitudinal myofibrils, generating radially symmetrical musculature similar to that found in polyp tentacles (Fig 3C). However, even in the distal constriction, endodermal cells never take on the “chordal” morphology seen in the polyp tentacle. Instead these cells are packed irregularly, often two cells thick (Fig 3D). These endodermal cells are smaller than those of the polyp, and lack the latter’s vacuolated morphology or circumferential fibers. Ectodermal musculature in the distal constricted portion of the medusa tentacle is also distinct from the polyp oral tentacle. Instead of contiguous longitudinal musculature, myofibrils appear to be organized into short bands of muscle restricted to modules revealed by Anti-Ttub staining (Fig 3E, 3F and 3G), which could represent cnidocyte batteries (see [19] for electron microscopy evidence of the same phenomena).

While the general neuroanatomy of oral and marginal tentacles appears similar, cnidocyte composition is distinct. At moderate magnification, FMRFamide and tryosinated tubulin-positive neural tracts are clearly visible in the bell, and extend into the tentacles (Fig 3H). At greater magnification, FMRFamide and Ttub-positive neurites from the diffuse sensory nerve net are visible innervating the musculature that radiates from the bell velum into the tentacle (Fig 3I, see [22] for similar results). The marginal tentacle ectoderm is rich in “capsule”-positive cnidocytes of a variety of sizes (Fig 3J). Again, this pattern is distinct from “capsule” staining in polyp tentacles (Fig 2K), where the antibody does not label cnidocyte capsules. TEM of the marginal tentacle reveals large numbers of microbasic heterotrichous euryteles (3L); we found no evidence for atrichous isorhizas, which are the dominant cnidocyte in oral tentacles. The distal dilation seen in the shaft of several fired “capsule”-positive cnidocytes (Fig 3K) is also consistent with descriptions of euryteles in *Aurelia* [27,28], and supports the antibody’s specificity to this cell type.

### EdU staining reveals different mechanisms of development between oral and marginal tentacles

**Fig 4:**
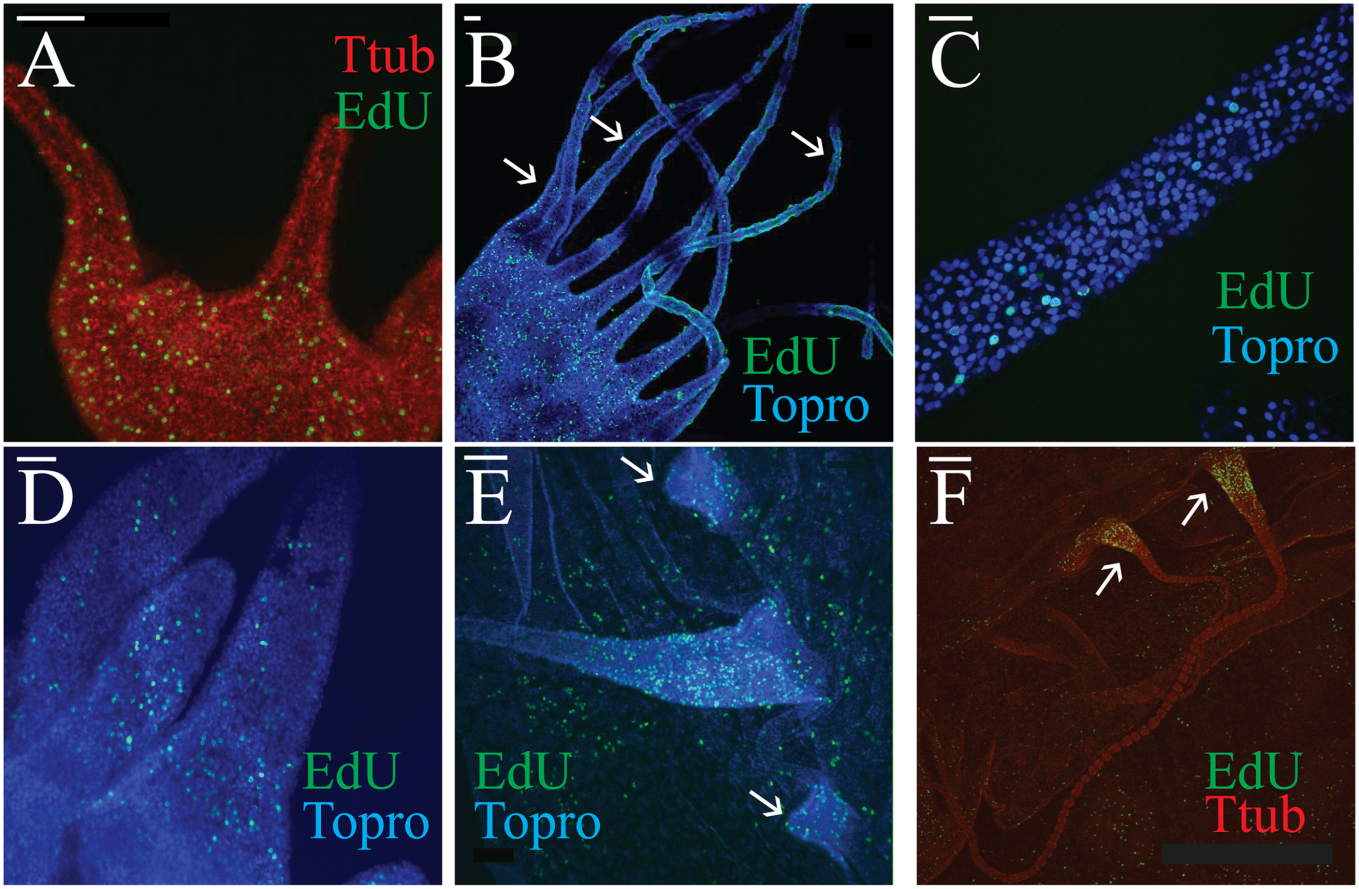
Cellular proliferation in *Aurelia sp*.*1* tentacles assayed with EdU. All samples were exposed to EdU for two hours before immediate fixation, and all scale bars equal 50 μm. (A) Developing tentacle of a young (primary) polyp. (B) Tentacles and the oral end of a mature polyp. EdU-positive cells are concentrated near the oral end of the animal, but are also found scattered across the tentacle ectoderm (a subset are marked with arrows). (C) A close-up of one polyp tentacle (approximately mid-length), elucidating the number and distribution of EdU-positive ectodermal cells. (D) Tentacles formed in a strobila undergoing regeneration during its conversion back into a polyp. The ephyrae attached to the strobila were cut away for clarity. (E) EdU proliferation in marginal tentacle buds (arrows) and a more developed tentacle (middle) from a young medusa. Notice how cellular proliferation in the more mature tentacle tapers off from the proximal (right) to distal (left) end. (F) Several marginal tentacles in an immature medusa, illustrating the band of EdU-positive cells at the proximal end (arrows).

To study cellular proliferation in *Aurelia* tentacles, we subjected live animals to two-hour incubations in EdU, a marker of S-phase cells. In the polyp, cellular proliferation of epitheliomuscular cells is constant, even in fully-grown individuals [29,30]. In the polyp tentacle specifically, cell division is continual and scattered in the ectoderm. This pattern remains consistent during the embryological growth of the tentacle (Fig 4A; and see [21]), homeostasis in mature polyps (Fig 4B), and during regeneration of the tentacle following strobilation (Fig 4C). While EdU staining was not observed in the tentacle endoderm, mitosis has been observed in previous studies [21]; this is consistent with the observation that the rate of cellular proliferation in *Aurelia’s* endoderm appears to be lower than in the ectoderm [29]. In the medusa, cellular proliferation in immature marginal tentacle buds is diffuse in both the ectoderm and endoderm (Fig 4E, arrows). But as the tentacle lengthens, S-phase cells in both germ layers become restricted to a band in the proximal portion (Fig 4E and 4F). This suggests that lengthening of the marginal tentacle occurs exclusively through cell proliferation at the tentacle’s base, which is distinct from the diffuse proliferation found in the oral tentacle.

## Discussion

This study provides evidence for significant morphological and developmental differences between polyp and medusa tentacles in *Aurelia sp*.*1*. The polyp tentacle—which develops in sets of four *via* scattered cellular proliferation—is a radially symmetrical structure of solid/chordal morphology, with coordinated, longitudinal musculature, and is rich in atrichous isorhizal cnidocytes. The medusa tentacle—which multifurcates and extends *via* a proximal growth zone—is bilaterally symmetrical, connected to the gastroderm, and features compartmentalized distal musculature and heterotrichous euryteles.

Much of this disparity is consistent with what is known about cnidarian tentacle diversity, and helps elucidate an emerging picture of cnidarian evolution. The solid tentacles seen in *Aurelia* polyps are considered a derived condition shared by scyphozoans, cubozoans, and some hydrozoans, and are understood as part of the simplification of the medusozoan polyp [7,10,31]. If the circular myofibrils we discovered are gastrodermal in origin, comparable to the chordal tentacles of *Hydractinia echinata* [32], then they might represent the remnants of more coordinated circular musculature found in the endoderm of hollow anthozoan tentacles, such as *Nematostella vectensis* [33]. Despite structural differences, *Aurelia* and *Nematostella* polyp tentacles both develop in sets of four, and exhibit cell proliferation marked by a punctate, uniform pattern [34]. In contrast, cell division is absent from the tentacles of *Hydra vulgaris* [35]; this likely represents a derived condition related to the canalization of *Hydra’s* growth to three stem cell populations restricted to the body column [30,36]. In the medusa, hollow tentacles also represent the probable ancestral condition [10], and it is possible that the unusual shape of *Aurelia’s* marginal tentacles are a property of hollow tentacles becoming distally constrained through their small size and high numbers. In the hydromedusa *Clytia hemisphaerica*, cell division and cnidogenesis is primarily restricted to interstitial stem cells in the proximal tentacle bulbs [37]. Assuming interstitial cells are a derived trait in hydrozoans [30,38], the proximal restriction of cellular division in *Aurelia* tentacles could represent the ancestral medusa condition, with growth restricted to the tentacle base, but prior to the evolution of interstitial stem cell lines. More work will be necessary to determine which cell types are dividing in the *Aurelia* marginal tentacle, and whether cnidogenesis is similarly concentrated in this region.

Still, not all of the differences between *Aurelia* tentacles can be readily explained through historical or morphological constraints, and some differences likely represent adaptive changes. For example, it is tempting to hypothesize that differences in musculature might result from differences in body muscle organization—with the medusa bell containing a circular band of coronal muscle and the polyp body column containing four longitudinal intramesogelal muscles. However, marginal tentacles in the jellyfish *Chrysaora quinquecirrha* feature radially symmetrical and uniform musculature, even though it contains similar coronal musculature to *Aurelia*, and develops from a nearly identical ephyra [39]. This suggests that the asymmetric musculature in the proximal marginal tentacle is adaptive. It is worth noting that the *Aurelia* polyp and medusa share a similar diet of zooplankton and other small metazoans [40], but capture their food in markedly different ways. When a polyp oral tentacle captures prey, it autonomously bends towards the mouth through retraction, and moves the food into the gastric cavity. Since we found no evidence of asymmetrical musculature within the oral tentacle, we concur with Chapman [17] that this behavior is not simply a byproduct of muscular biomechanics. Instead, each tentacle acts as a sensory appendage [41,42] actively involved in prey detection, capture, and manipulation. In the medusa, marginal tentacles are only part of the food-capturing process; predation is a consequence of zooplankton coming into contact with any part of the oral side of the animal as water passes across the pulsating bell [43]. The behavior of medusa tentacles has received little study beyond the classical work by Romanes [44], who demonstrated that a proper stimuli can elicit a wave of tentacle retractions. This suggests that, unlike the polyp, medusa marginal tentacles do not act autonomously. Instead the tentacles, which are highly ciliated, pass prey into the bell’s food groove, which is then picked up by the oral arms and moved into the stomach [19,45]. Thus, marginal tentacles appear to play a less dynamic role in prey capture compared to polyp tentacles, which could explain the former’s limited distal musculature. While the distal portion of the marginal tentacle follows flow vortices generated by the pulsing bell, aiding in the capture of prey [46], oral musculature keeps the proximal portion rigid in the face of vortices generated [19]. Such rigidness might aid in the swimming stroke mechanism by enhancing propulsion, or could improve osmotic uptake by thinning boundary layers at the tentacle’s surface [47].

In contrast to the hypotheses presented above, the differences in cell proliferation suggest that the two tentacle forms exhibit distinct extension mechanisms, and therefore lack an aspect of process-level homology [48] in growth and development. This observation challenges the hypothesis that oral and marginal tentacles are derived from a common tentacle precursor in a conserved cnidarian body plan. This *bauplan* hypothesis—a standard implicit or explicit conception in most invertebrate zoology textbooks [2,49–53]—argues that polyp and medusa tentacles exhibit a form of serial homology, representing structures with a shared evolutionary ancestry that are temporally recapitulated during the animal’s development. Despite the prevalence of this hypothesis, there are cases where tentacle-like structures likely evolved convergently within the *Cnidaria*. For example, the hydrozoan genus *Obelia* appears to have independently re-evolved a medusa life-stage, with solid tentacles as opposed to the hollow form typical of hydromedusa [54]. Additionally, *Hox* gene expression from *Hydractinia* dactylozooids suggests that these “tentacles” are derived from an elongated hydroid body column, with the head and “true” oral tentacles retarded during development [55]. Comparative genetic data on tentacle morphogenesis should help resolve their homology, but such evidence is currently lacking, even amongst the model cnidarians. Transcription factors required for tentacle formation in *Hydra* (such as *aristaless* [56]) have not been studied in the anthozoan model system *Nematostella*, and the opposite is true of genes strongly expressed in *Nematostella* tentacles (such as certain *Pax* domain genes [57]). While both *Nematostella* and *Hydra* require Wnt/β-Catenin signaling for proper tentacle formation [58,59], this could simply be a function of the pathway’s role in broadly controlling epithelial evaginations [60]. Future molecular genetic research into *Aurelia* and other non-model systems, alongside traditional cnidarian model systems such as *Hydra* and *Nematostella*, should prove valuable for clarifying the evolution and homology of cnidarian tentacles.
